# Overcoming Resistance of Caco-2 Cells to 5-Fluorouracil through Diruthenium Complex Encapsulation in PMMA Nanoparticles

**DOI:** 10.1021/acs.inorgchem.4c01323

**Published:** 2024-06-04

**Authors:** Isabel Coloma, Jorge Parrón-Ballesteros, Miguel Cortijo, Cristián Cuerva, Javier Turnay, Santiago Herrero

**Affiliations:** † MatMoPol Research Group, Inorganic Chemistry Department, Faculty of Chemical Sciences, Complutense University of Madrid, E-28040 Madrid, Spain; ‡ Department of Biochemistry and Molecular Biology, Faculty of Chemical Sciences, Complutense University of Madrid, E-28040 Madrid, Spain

## Abstract

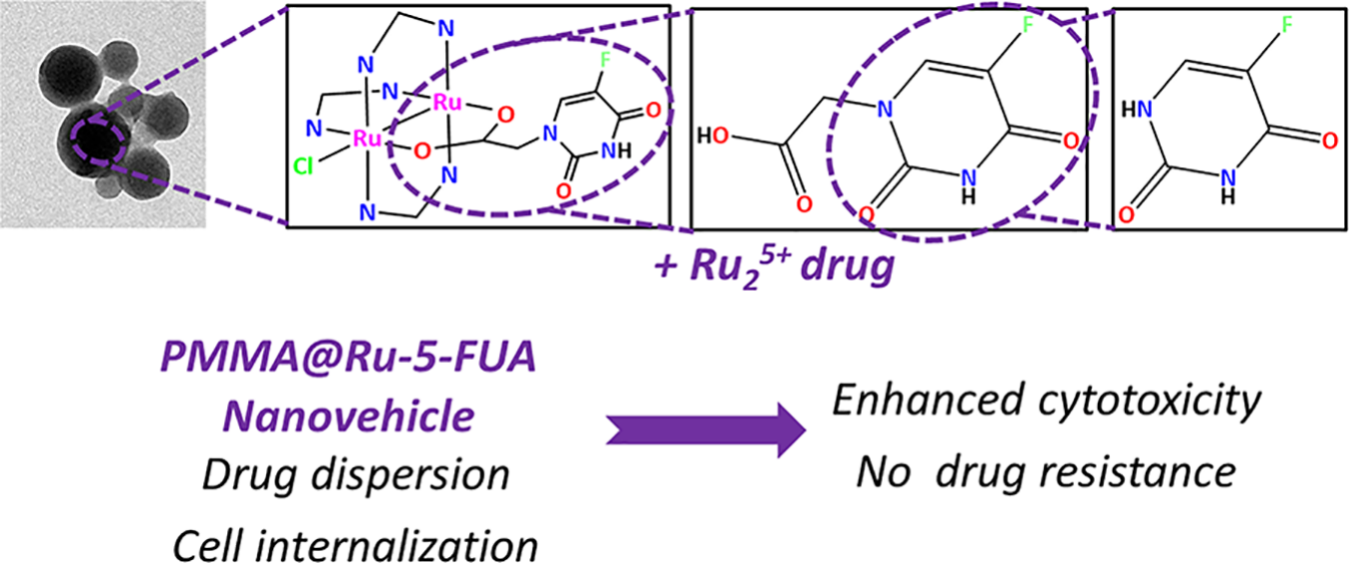

Drug resistance, one of the main drawbacks in cancer chemotherapy, can be tackled by employing a combination of drugs that target different biological processes in the cell, enhancing the therapeutic efficacy. Herein, we report the synthesis and characterization of a new paddlewheel diruthenium complex that includes 5-fluorouracil (5-FU), a commonly used anticancer drug. This drug was functionalized with a carboxylate group to take advantage of the previously demonstrated release capacity of carboxylate ligands from the diruthenium core. The resulting hydrophobic complex, [Ru_2_Cl(DPhF)_3_(5-FUA)] (**Ru-5-FUA**) (DPhF = *N*,*N*′-diphenylformamidinate; 5-FUA = 5-fluorouracil-1-acetate) was subsequently entrapped in poly(methyl methacrylate) (PMMA) nanoparticles (**PMMA@Ru-5-FUA**) via a reprecipitation method to be transported in biological media. The optimized encapsulation procedure yielded particles with an average size of 81.2 nm, a PDI of 0.11, and a zeta potential of 29.2 mV. The cytotoxicity of the particles was tested in vitro using the human colon carcinoma cell line Caco-2. The IC_50_ (half maximal inhibitory concentration) of **PMMA@Ru-5-FUA** (6.08 μM) was just slightly lower than that found for the drug 5-FU (7.64 μM). Most importantly, while cells seemed to have developed drug resistance against 5-FU, **PMMA@Ru-5-FUA** showed an almost complete lethality at ∼30 μM. Conversely, an analogous diruthenium complex devoid of the 5-FU moiety, [Ru_2_Cl(DPhF)_3_(O_2_CCH_3_)] (**PMMA@RuA**), displayed a reduced cytotoxicity at equivalent concentrations. These findings highlight the effect of combining the anticancer properties of 5-FU with those of diruthenium species. This suggests that the distinct modes of action of the two chemical species are crucial for overcoming drug resistance.

## Abstract 2

Drug resistance, one of the main drawbacks in cancer chemotherapy, can be tackled by employing a combination of drugs that target different biological processes in the cell, enhancing the therapeutic efficacy. Herein, we report the synthesis and characterization of a new paddlewheel diruthenium complex that includes 5-fluorouracil (5-FU), a commonly used anticancer drug.

## Introduction

The use of chemotherapeutic agents, alone or combined with other types of therapy, is the most widely used approach for cancer treatment. However, most anticancer drugs induce several side effects such as myelosuppression, neurotoxicity, or nephrotoxicity. In addition, chemotherapy may be unsuccessful due to acquired drug resistance, which can be achieved, for instance, by increased levels of glutathione, thioredoxin reductase, antiapoptotic proteins, xenobiotic membrane pumps, or DNA damage repair proteins.^1^

The existence of the two above-mentioned limitations keeps encouraging an intense search and screening for novel pharmacological agents. Among classical anticancer drugs, those based on platinum complexes, i.e., cisplatin and related compounds, have been employed in the clinical treatment of several types of cancers, owing to their high activity and economic affordability. However, their usefulness is still constrained because of drug resistance issues and severe side effects, which are dose-limiting.^2−4^ Some ruthenium-based drugs emerged as alternatives with lower toxicity than platinum derivatives.^5,6^ In particular, NAMI-A, KP1019, (N)KP1339, TLD1433, and RAPTA-C managed to enter clinical trials phases I and II.^7−11^ Their remarkable activity has been ascribed to a rate of ligand exchange on the same scale of cell lifetimes,^5,12^ different biologically accessible oxidation states,^13^ and even to their ability to mimic iron.^12,14^ It has also been observed that the cytotoxicity of the mononuclear ruthenium complex [Ru(bpy)(5-FU)(PPh_3_)_2_](PF_6_) is much higher than the anticancer drug 5-FU alone while the parent compound [Ru(bpy)Cl_2_(PPh_3_)_2_] shows only a weak cytotoxicity.^15^

A family of related complexes, which has been less explored in this field, is the one formed by diruthenium compounds with paddlewheel structure despite their anticancer properties were discovered already three decades ago.^16,17^ These complexes are formed by two metal–metal-bonded ruthenium atoms, usually both with an average oxidation state of 2.5, bridged by four equatorial bidentate ligands and usually containing one axial ligand as well. The axial ligands of these complexes are usually quite labile, but carboxylate equatorial bridging ligands can also be removed, especially in slightly acidic media, to provide a spatial distribution of coordination vacancies in the metals that make them able to interact with other chemical species in a way impossible to achieve by any mononuclear compound. Moreover, the strong metal–metal bond facilitates cooperation between the ruthenium centers during the ligand exchange reactions.

It has been observed that the coordination of naproxen or ibuprofen through their carboxylate groups to a paddlewheel diruthenium unit leads to complexes that substantially reduce the proliferation of C6 rat and human glioma cells.^18,19^ Moreover, the encapsulation of some of these complexes in solid polymer–lipid nanoparticles or chitosan gives rise to enhanced cytotoxicity in breast (EMT6 and MDA-MB-231),^20^ prostate (DU145),^20^ and glioma cancer cells.^21,22^ Another recent contribution has shown that two isomeric diruthenium complexes containing indolylglyoxylyl dipeptides display a remarkably different behavior against a glioblastoma model.^23^ How these diruthenium compounds exert their antitumor activity is largely unknown, and only a few studies showed how they interact with biological molecules. Particularly, reactions with ascorbic acid or glutathione have been studied from a kinetic and mechanistic point of view,^24^ as well as the coordination to amino acids,^25^ proteins,^26−63^ nucleotides,^33^ or even RNA.^34^

It has also been recently reported that the presence of three bridging formamidinate ligands provides special stability to the diruthenium moiety, and these triformamidinato-Ru_2_^5+^ complexes can be valuable platforms for the development of drug carriers. The controlled release of bioactive carboxylate ligands,^35,36^ such as 2,4-dichlorophenoxyacetate (2,4-D) or 1-naphthaleneacetate (NAA), has been determined by quantitative in vivo studies performed using transgenic *Arabidopsis thaliana* plants.^35^ The conclusion of those studies is that carboxylate bridging ligands in paddlewheel diruthenium compounds are slowly released at physiological pH but rapidly in slightly acidic media.

In the present work, we exploit the above-mentioned characteristics coordinating an antitumor prodrug to a diruthenium core through a carboxylate group. The selected ancillary ligand is *N*,*N*′-diphenylformamidinate (DPhF), and the chosen prodrug 5-fluorouracil-1-acetate (5-FUA), leading to the complex [Ru_2_Cl(DPhF)_3_(5-FUA)] (**Ru-5-FUA**). Once liberated from the diruthenium complex, 5-FUA suffers a decarboxylation process forming 5-fluorouracil (5-FU) species in the cytosol.^37−39^ 5-FU is a drug commonly employed in the clinical treatment of several types of tumors, mainly for the treatment of breast and colorectal cancer. Its mechanism of action is based on the inhibition of thymidylate synthase and the incorporation of its metabolites into DNA and RNA. However, it causes the development of drug resistance that can be reduced or abrogated if the metal centers of this type of compounds exert an additional cytotoxic effect through a different mode of action.

Polymer particles have been prepared to encapsulate the hydrophobic **Ru-5-FUA** and serve as nanocarriers for the compound in biological media and to facilitate its internalization into cells. Nanoparticles have been obtained via the reprecipitation method, using poly(methyl methacrylate) (PMMA) as a biocompatible polymer.^40,41^ In order to evaluate the usefulness of the new Ru_2_^5+^-doped polymer nanoparticles in biomedical applications, in vitro studies have been carried out using as a cell model system the undifferentiated human colorectal adenocarcinoma cell line Caco-2, as it is one of the most widely used cell lines for the analysis of the effect of cytotoxic compounds for their potential use in chemotherapy. In addition, this cell line is quite heterogeneous, presenting different subpopulations and the ability to differentiate into different cell subtypes. More interestingly, these cells present radio and chemoresistance to different agents, including 5-FU, which makes them an ideal model system for these studies.^42,43^

For comparison, those studies have also been carried out with polymer nanoparticles without diruthenium compounds, as well as pure 5-FU and other particles loaded with an analogous diruthenium complex, [Ru_2_Cl(DPhF)_3_(O_2_CCH_3_)] (**PMMA@RuA**), without the 5-FU drug. These control experiments have been carried out to study the potentially enhancing effect of the diruthenium species with respect to 5-FU, and to corroborate that the nanovehicles are biocompatible and do not exhibit cytotoxicity by themselves. The internalization of diruthenium complexes has been studied by confocal fluorescence microscopy using PMMA nanoparticles that contain coumarin 6 as a fluorescence probe.

## Results and Discussion

### Synthesis and Characterization of **Ru-5-FUA**

**Scheme 1:**
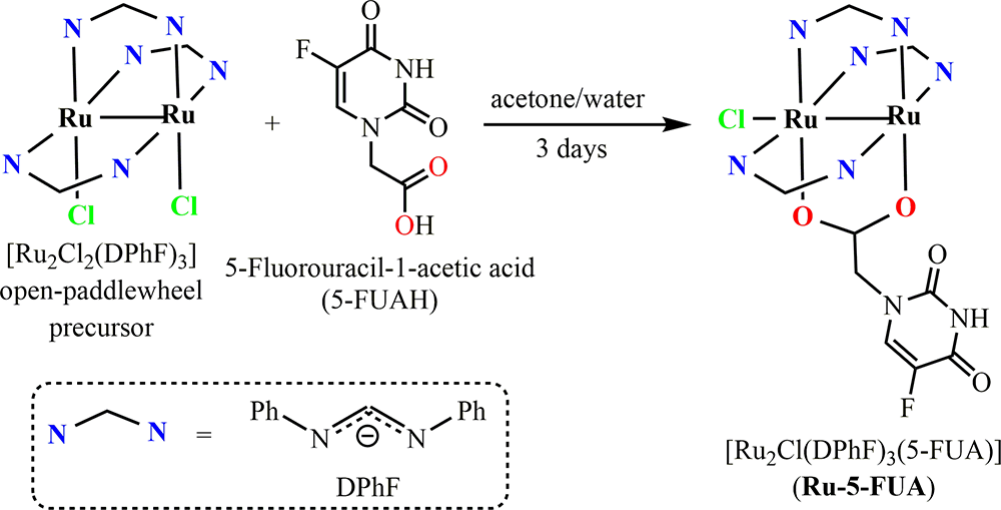
Schematic Synthesis of [Ru_2_Cl(DPhF)_3_(5-FUA)] (**Ru-5-FUA**) (DPhF = *N*,*N*′-Diphenylformamidinate; 5-FUA = 5-Fluorouracil-1-acetate)

The preparation of **Ru-5-FUA** was carried out following a previously reported synthetic strategy for the coordination of carboxylate-containing bioactive molecules to the diruthenium core (see Scheme 1), using [Ru_2_Cl_2_(DPhF)_3_] as a precursor.^35,36^ The use of this reactive species with an open-paddlewheel structure allows the coordination of the 5-fluorouracil-1-acetate (5-FUA) ligand through its carboxylate group under mild reaction conditions and acidic media. Otherwise, coordination would probably be through the uracil group.

Mass spectrometry data suggest the isolation of a complex with a 1:1 diruthenium-ligand stoichiometry, as a single significant peak (*m*/*z* = 976.179), corresponding to a [M-Cl]^+^ fragment (*m*/*z* = 976), was observed (see Figures S1 and S2). In addition, the ^19^F-NMR spectrum shows a unique signal at −168.75 ppm, confirming the formation of a single product containing the 5-FUA ligand.

Infrared spectroscopy data suggest the coordination of 5-FUA through the carboxylate group with a bridging coordination mode. Two intense bands are observed around 1520 and 1425 cm^–1^ (see Figure S3), which can be attributed to the antisymmetric and symmetric O–C–O stretching bands, respectively.^35,36^ Moreover, the separation between these two bands suggests the aforementioned bridging coordination mode.^44^ Also, two signals can be observed in the spectrum around 3175 and 1700 cm^–1^, which correspond to N–H and C=O stretching modes of the ligand 5-FUA.

Moreover, the electronic spectrum in dichloromethane solution (see Figure S4), shows the typical electronic profile of [Ru_2_Cl(DPhF)_3_(carboxylate)] compounds.^36,45,46^ The spectrum displays one maximum and several shoulders. The maximum at 523 nm and the shoulder at ∼571 nm can be ascribed to π*(Ru2) → σ*(RuN) and π*(Ru2) → σ*(RuO). The shoulder at ∼641 nm is associated with a δ(Ru2) → π*(Ru2) transition.^47^

**Figure 1:**
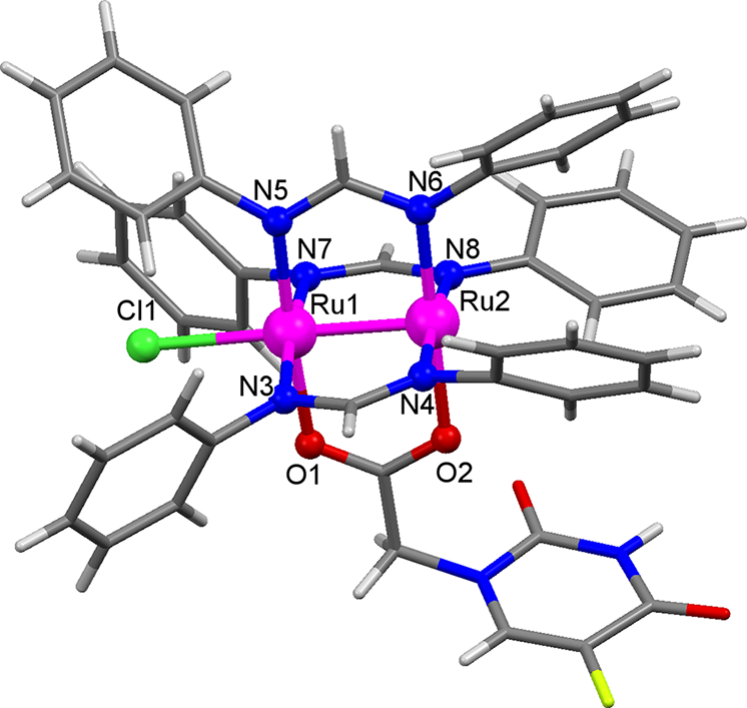
View of the asymmetric unit of **Ru-5-FUA**·0.5THF with selected atoms labeled. Ru atoms are shown in pink, N atoms in blue, O atoms in red, Cl atom in green, C atoms in gray, F atoms in yellow, and H atoms in white. Crystallization solvent molecules have been omitted for clarity.

**Figure 2:**
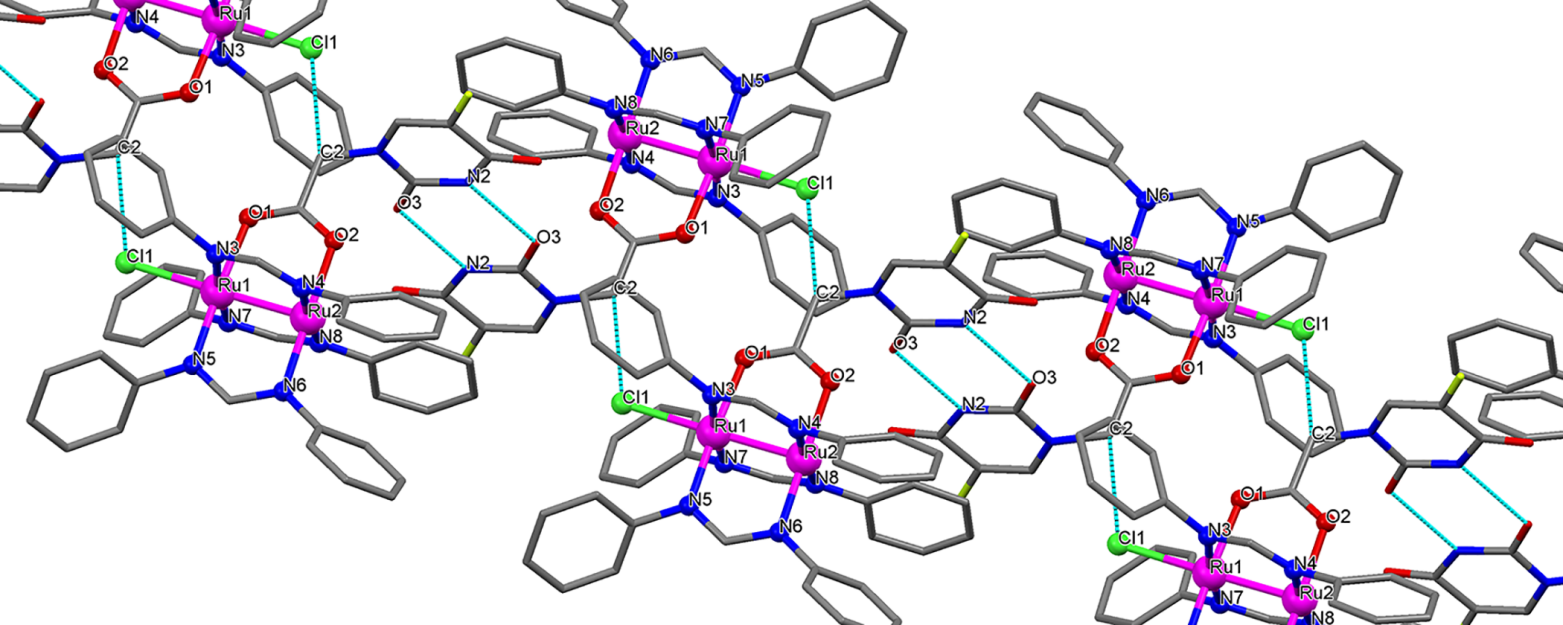
Intermolecular interactions found in the crystal structure of **Ru-5-FUA**·0.5THF.

All of the previous statements were confirmed by single crystal X-ray diffraction (see Table S1 for crystal and structure refinement data). Single crystals of **Ru-5-FUA**·0.5THF were obtained by slow diffusion of hexane in a THF solution of the complex. The crystal structure confirms the presence of a diruthenium compound with a paddlewheel structure, where the two metal atoms are supported by three formamidinate bridging ligands, and 5-FUA coordinated through the carboxylate group as the fourth bidentate ligand. The complex also presents a chloride ligand at one of the axial positions. The most relevant bond distances and angles are given in Table S2. Ru1–Ru2 bond distance is 2.312 Å, which corresponds to a bond order of 2.5. Figure 1 shows the asymmetric unit of **Ru-5-FUA**·0.5THF. In Figure 2, one C–H···Cl intermolecular weak interaction between Cl1 and C2 (3.238 Å) and intermolecular hydrogen bonds involving N2 and O3 from the ligand 5-FUA of two diruthenium units (2.809 Å) are shown.

### Preparation and Characterization of PMMA@Ru-5-FUA Nanoparticles

The encapsulation of hydrophobic **Ru-5-FUA** in polymer particles was successfully performed via the reprecipitation method,^40,41^ by using a THF solution of the diruthenium compound and PMMA as the oil phase, and water. PMMA was used as the polymer matrix based on both its excellent colloidal stability and its good biocompatibility.^48^ Moreover, it has been demonstrated that PMMA nanoparticles show a certain selective effect toward colon cancer cells.^49^ As depicted in Figure S5, oil-in-water (O/W) droplets containing **Ru-5-FUA** and PMMA were obtained through two different synthetic pathways. In a direct way, by adding the oil phase over water (method 1), or by means of an intermediate stage that originates inverse water-in-oil emulsions under the addition of water over the oil phase (method 2). After the droplet stabilization process at 20 °C, THF evaporation under vacuum gives rise to colloidal dispersions of the desired **Ru-5-FUA**-loaded PMMA particles in water.

The colloidal dispersions obtained from both methods were studied by dynamic light scattering (DLS) at 25 °C. Table 1 collects the Z-average size, polydispersity index (PDI), and zeta potential values for all particles synthesized. Results clearly evidence that the direct formation of the O/W microemulsions (method 1) originates smaller polymer particles (Z-average: 529.2 nm) than those prepared from method 2 (Z-average 3.1 μm). As expected, the transformation of the inverse W/O microemulsions into O/W microemulsions by increasing the water content hinders the control over the particle size. In fact, larger polymer fibers and precipitates can be observed with the naked eye when method 2 is used.

**Table 1:** Summary of Particle Characterization Data

	**conditions**	**Z-average**^a^**/ nm**	**PDI**^a^	**zeta potential**^a^**/ mV**
method 1	fast THF evap./20 °C	529.2	0.082	0
	slow THF evap./20 °C	127.8	0.18	37.6
	slow THF evap./4 °C	176.9^b^	0.31	35.1
method 2	fast THF evap./20 °C	3147	0.38	

a DLS data measured at 25 °C.

b Bimodal size distribution with mean values of ca. 80 and 380 nm.

**Figure 3:**
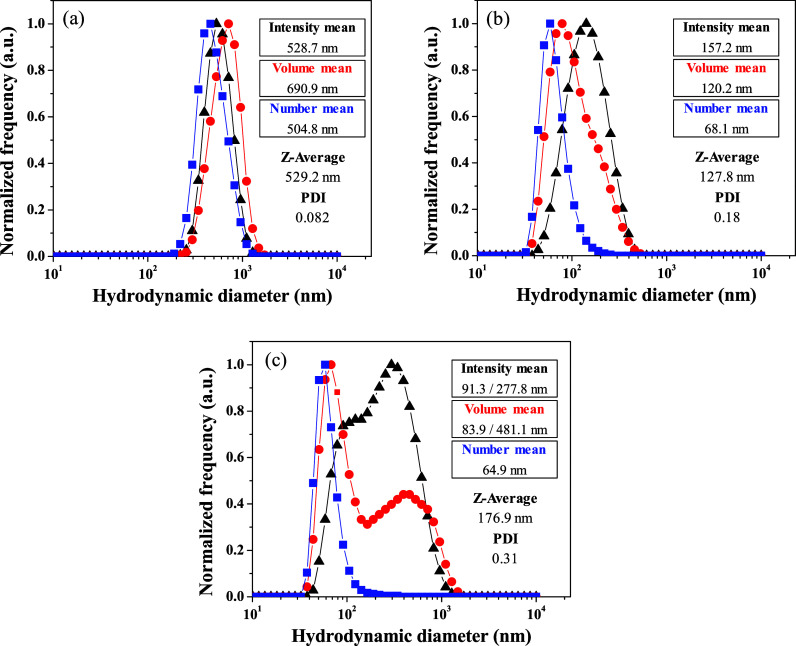
DLS analysis for **PMMA@Ru-5-FUA** particles obtained via method 1 under (a) droplet stabilization at 20 °C and a fast THF evaporation process, (b) droplet stabilization at 20 °C and a slow THF evaporation process, and (c) droplet stabilization at 4 °C and a slow THF evaporation process.

To further analyze the effect of the reaction conditions on the particle size and concomitantly optimize the synthetic procedure, several aqueous dispersions of particles were prepared via method 1 by varying the evaporation rate of THF as well as the temperature of the droplet stabilization process. In the first case, slow evaporation of THF at room temperature for 24 h allowed reduction of the distortions in the microemulsions during the formation of particles, decreasing the Z-average size from 529.2 to 127.8 nm (Figure 3a, b). Moreover, the zeta potential value was found to be 37.6 mV, which suggests that this new synthetic procedure improves the colloidal stability of the particle dispersion. Regarding the PDI, it slightly increases from 0.082 to 0.18 (Table 1).

On the other hand, it is well-known that the Brownian motion in a colloidal system decreases at lower temperatures, and subsequently the coalescence processes, which may originate smaller particles than those prepared at room temperature. Keeping in mind this fact, PMMA particles were also prepared via method 1 by stabilization of the oil-in-water microemulsions at 4 °C for 24 h, followed by slow THF evaporation at ambient temperature. As shown in Figure 3c, a bimodal size distribution is obtained from DLS analysis with mean values of about 80 and 380 nm. The presence of a unique number size distribution with a mean value of 64.9 nm suggests that the smallest particles are the majority, as expected by decreasing the temperature of the droplet stabilization. However, coalescence processes continue to occur, and some small particles are merged with each other to yield larger particles of the order of 380 nm (intensity mean: 277.8 nm; volume mean: 481.1 nm). This makes the colloidal dispersions prepared by this method not monodisperse (PDI value of 0.31), the average particle size and zeta potential being 176.9 nm and 35.1 mV, respectively (Table 1).

**Figure 4:**
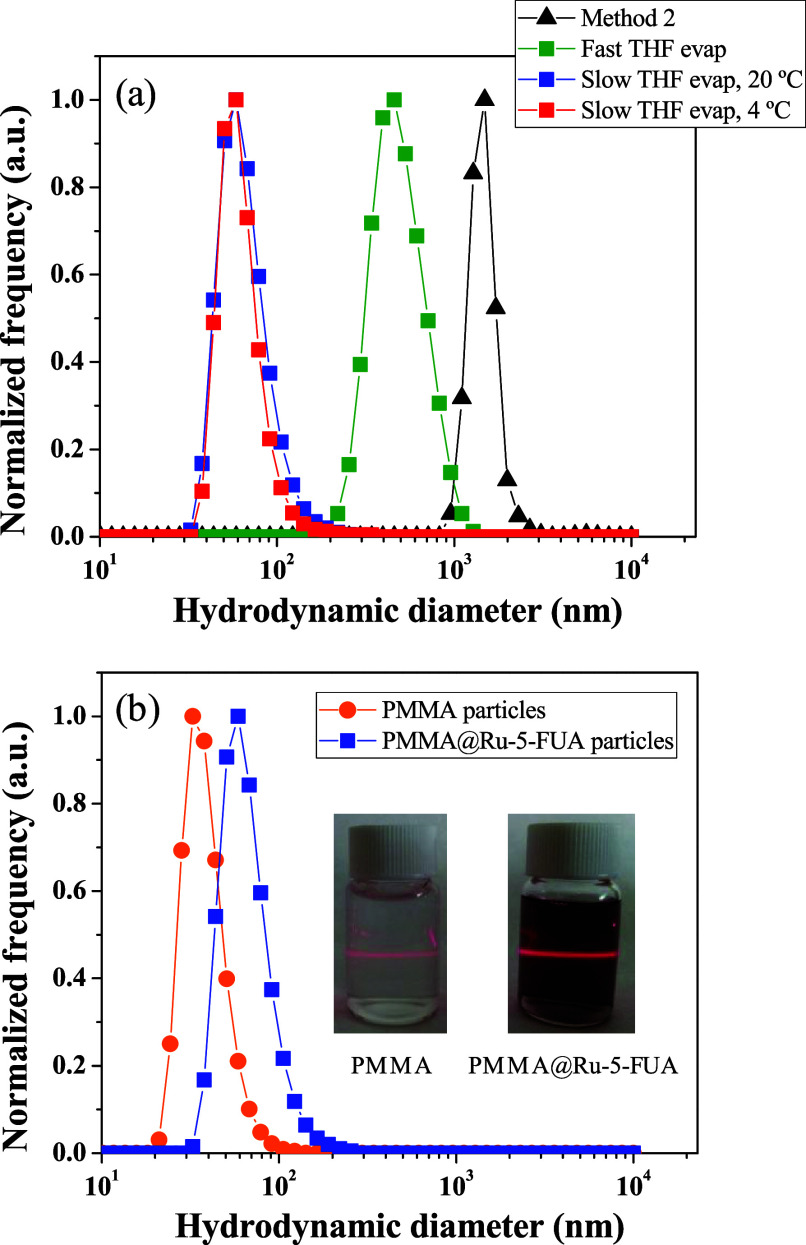
(a) DLS number size distributions for the **PMMA@Ru-5-FUA** particles prepared via method 1 (squares) under different conditions and method 2 (triangles). (b) DLS spectra registered for nonloaded PMMA and **PMMA@Ru-5-FUA** particles obtained via method 1 under droplet stabilization at 20 °C, followed by slow THF evaporation at room temperature. The inset displays images of both aqueous dispersions, showing the Tyndall effect.

Figure 4a shows selected DLS number size distributions registered for each aqueous dispersion of **PMMA@Ru-5-FUA** particles for comparative purposes. Results clearly evidence that method 1 with slow THF evaporation processes is the best synthetic procedure to prepare well-dispersed and smaller particles. In particular, when droplet stabilization occurs at 20 °C, highly stable and monodisperse colloidal systems can be obtained. The O/W microemulsions act as a template, driving the growth of the polymer particles during the oil evaporation process, which in turn trap the diruthenium compound inside.

This method was also used to prepare nonloaded PMMA particles under the same conditions. As observed in Figure 4b, the entrapment of **Ru-5-FUA** only causes a slight increase in the average particle size from 77.4 to 127.8 nm, as expected, along with an easy-to-notice color change. Moreover, it is also interesting to remark that no precipitate of **Ru-5-FUA** is observed in the aqueous dispersion either when it is synthesized or during subsequent weeks, despite the hydrophobic nature of the diruthenium derivative. All of these features indicate that the entrapment of **Ru-5-FUA** inside the PMMA particles is successful under the above-mentioned conditions.

### PMMA@Ru-5-FUA Nanoparticles for Cell Cytotoxicity Assays

Based on the above-described encapsulation studies, **PMMA@Ru-5-FUA** nanoparticles were prepared via method 1 by using Milli-Q water. Droplet stabilization was carried out at 20 °C for 24 h, and THF was allowed to slowly evaporate during a subsequent period of 24 h. Note that this procedure showed the best results in terms of particle size, monodispersity, and colloidal stability.

**Figure 5:**
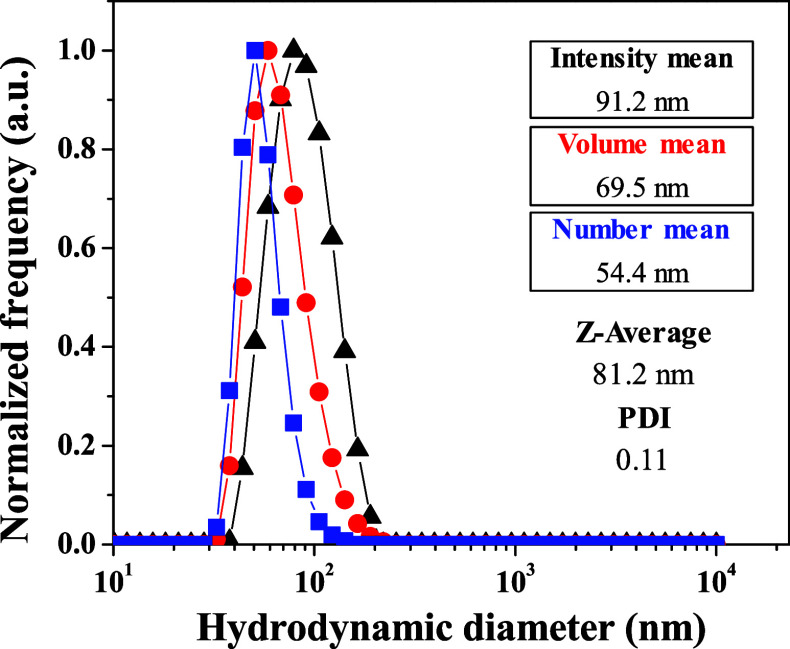
DLS analysis for the aqueous dispersion of **PMMA@Ru-5-FUA** nanoparticles prepared in ultrapure Milli-Q water.

**Figure 6:**
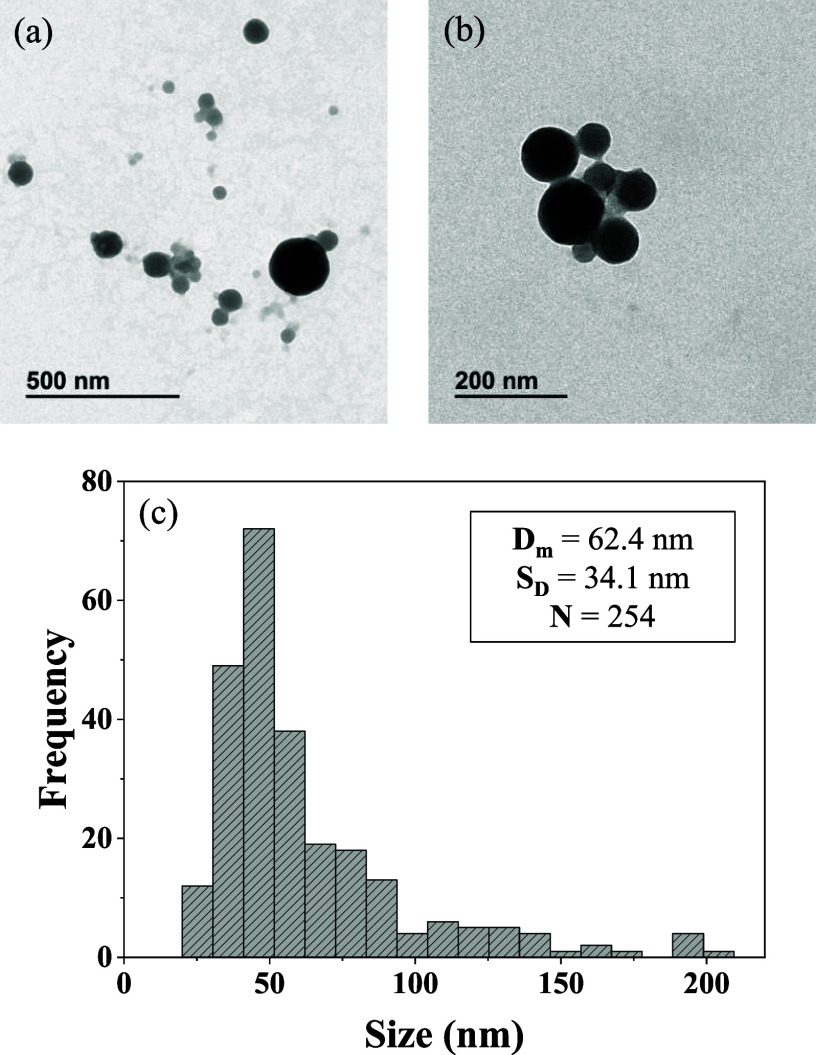
(a, b) TEM microphotographs showing the **PMMA@Ru-5-FUA** nanoparticles. (c) Particle size distribution histogram. The average diameter (*D*_m_), standard deviation (*S*_D_) and number of particles (*N*) used for TEM analysis are also indicated.

As expected, a unique size distribution is observed from DLS spectra (Figure 5); the new nanoparticles show an average hydrodynamic diameter of 81.2 nm, a PDI value of 0.11, and a zeta potential value of 29.2 mV. It is noticeable that both the particle size and PDI values decrease with respect to those found previously, so the use of ultrapure Milli-Q water favors the stabilization of the O/W microemulsions before the formation of particles. Transmission electron microscopy (TEM) analysis was also performed to study the morphology of the particles. Results confirm that the O/W microemulsions prepared by the droplet method allow us to obtain spherical polymer nanoparticles (Figure 6a,b). In addition, the average diameter was calculated to be 62.4 nm (Figure 6c), which is consistent with the hydrodynamic diameter measured by DLS. With regard to the entrapment of **Ru-5-FUA** into the PMMA particles, it occurs again successfully, achieving an entrapment efficiency of 62%, as determined by UV–vis spectroscopy. Visual aggregation or precipitation has not been observed over time. In fact, the particle size and the PDI values were monitored for three months, and no drastic changes were recorded, which demonstrates high colloidal stability (Figure S6).

### Cell Viability Assays

**Figure 7:**
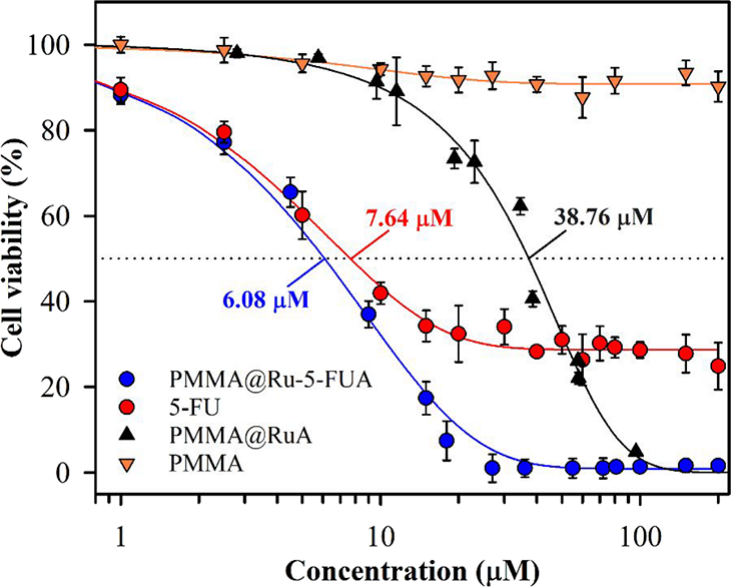
Semilogarithmic representation of Caco-2 cell viability after 72 h of incubation in the presence of the indicated compounds. IC_50_ values are indicated. Values corresponding to 5-FU and **PMMA@Ru-5-FUA** are the average ± SD of 5 different assays whereas PMMA and **PMMA@RuA** was only assayed twice.

Undifferentiated Caco-2 cells were cultured in 24-well plates under standard culture conditions for 48 h prior to the addition of serial dilutions of **PMMA@Ru-5-FUA** and 5-FU. The complex [Ru_2_Cl(DPhF)_3_(O_2_CCH_3_)] (RuA), a diruthenium complex that does not contain 5-FUA, was also synthesized and encapsulated into PMMA nanoparticles (**PMMA@RuA**) to assess the activity of the diruthenium species alone; in addition, controls of PMMA nanoparticles at equivalent concentration were used. These assays showed that **PMMA@Ru-5-FUA** presents an IC_50_ cytotoxicity value in the low micromolar range. Thus, cell viability assays were carried out using concentrations of the complex from 1 μM up to 200 μM in comparison with 5-FU, **PMMA@RuA**, and nude PMMA nanoparticles at equivalent concentrations as those used with the diruthenium complex (Figure 7).

As expected, empty PMMA nanoparticles induced only a minor decrease (less than 10%) in cell viability after 72 h incubation, which could arise from either mechanical stress or energy consumption due to endocytosis of the nanoparticles. On the other hand, both **PMMA@Ru-5-FUA** and 5-FU induced a potent cytotoxic effect with IC_50_ values of 6.08 ± 0.27 and 7.64 ± 0.20 μM, respectively. Interestingly, although IC_50_ values are quite similar, the decrease in cell viability induced by 5-FU stabilizes at 28.7 ± 0.7% and did not go further down probably due to the development of drug resistance or, more probably, to the preexistence of drug-resistant Caco-2 cell subpopulations. In contrast, **PMMA@Ru-5-FUA** achieves an almost complete lethality at ∼30 μM avoiding drug resistance. Nanoparticles containing the diruthenium compound without 5-FU (**PMMA@RuA**) presented a significantly higher IC_50_ value (38.76 ± 1.47 μM) but were able to induce almost 100% lethality at concentrations above 100 μM. Thus, it looks like cytotoxicity at low concentrations of **PMMA@Ru-5-FUA** is mainly due to the release of 5-FUA, whereas at slightly higher concentrations, the diruthenium core adds its own cytotoxic effect to that of 5-FUA, overcoming cell chemoresistance.

Other human colorectal carcinoma cells have been reported to present higher IC_50_ values for 5-FU (>20 μM) and could be considered as more resistant to 5-FU than Caco-2 cells, but all these cell lines present very low viability around 100 μM 5-FU,^50^ in contrast to what we have observed for Caco-2 cells. This strongly suggests the presence of a highly 5-FU resistant cell subpopulation in this cell line in addition to other cells that are more sensitive to this agent. Thus, Caco-2 cells constitute an ideal model for testing new drugs to overcome resistance to novel chemotherapeutic formulations.

**Figure 8:**
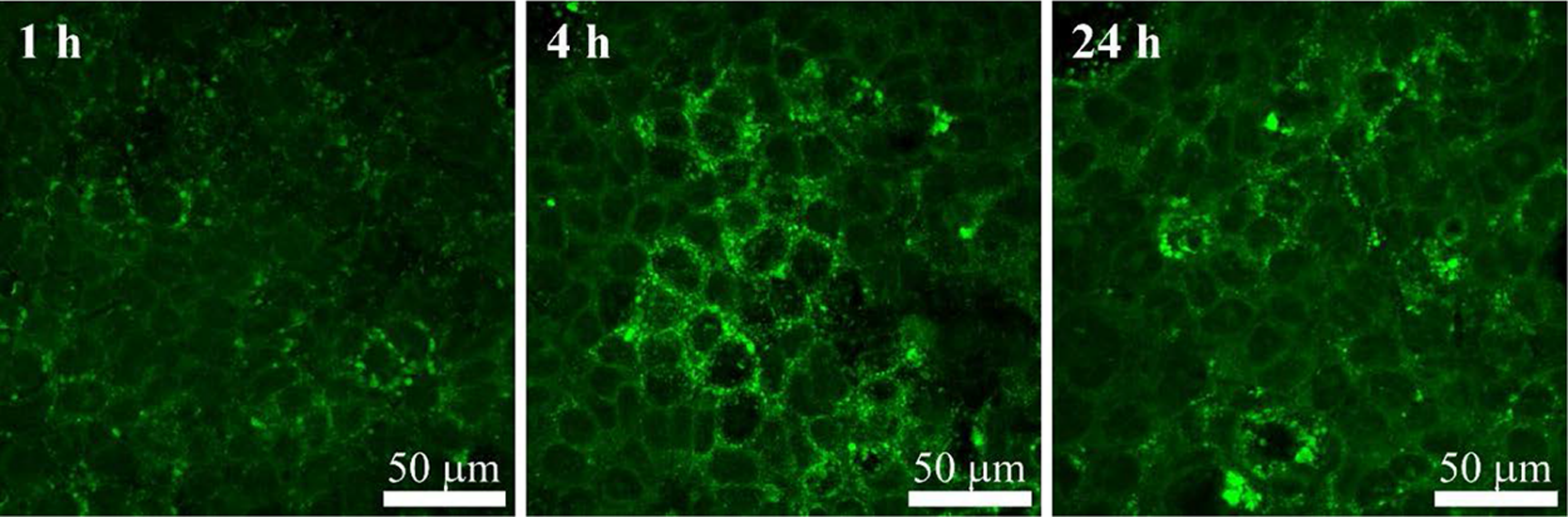
Confocal images of Caco-2 cells exposed to PMMA nanoparticles containing coumarin 6 after 1, 4, and 24 h.

5-FU can permeate Caco-2 cell membranes by passive diffusion^51^ to exert its intracellular effects by inhibition of thymidylate synthase and incorporation into nucleic acids. However, Caco-2 cells, as well as many other malignant cells, are known to express several drug-metabolizing enzymes and transporters, including cytochromes P450, carboxylesterases, and efflux transporters such as P-glycoprotein^51,52^ which could be responsible for the above-mentioned drug-resistance. On the other hand, PMMA nanoparticles enter cells via endocytosis, even at short incubation times. Since **Ru-5-FUA** does not exhibit fluorescence emission even when excited at its absorption maximum, this feature has been verified by confocal microscopy using emissive coumarin 6-loaded nanoparticles (Figure 8), already described for other cell types.^53^

Endocytosis may drive **PMMA@Ru-5-FUA** toward acidic lysosomes where 5-FUA will be released. The release of carboxylate ligands from triformamidinato-Ru_2_^5+^ units under acidic conditions was previously demonstrated.^35,36^ Afterward, 5-FUA can be transformed into 5-FU.^37−39^ In addition, the remaining diruthenium core is also cytotoxic, as shown with **PMMA@RuA** control experiments, adding its effects to those of 5-FU and avoiding the appearance of resistance.

## Conclusions

A new diruthenium complex functionalized with 5-fluorouracil-1-acetate (**Ru-5-FUA**) was synthesized as a potential cytotoxic drug. Although the complex is not soluble in water, a direct reprecipitation method has been successfully used to achieve the entrapment of **Ru-5-FUA** into PMMA polymer particles. The control of the reaction conditions and the synthetic method allow modulating the size of the oil-in-water droplets and, concomitantly, the hydrodynamic diameter of the final loaded particles in the size range of micro and nanoscale. It is demonstrated in this work that the particles act as nanocarriers of the complex in the culture medium, achieving its internalization inside the Caco-2 cells. The in vitro cytotoxicity assays presented herein demonstrate that the use of **PMMA@Ru-5-FUA** prevents the development of drug resistance observed when 5-FU is employed alone. This suggests, first, that **Ru-5-FUA** releases the prodrug 5-FUA inside the cells, where the active 5-FU species are formed. Second, the resulting diruthenium moiety also exhibits anticancer activity and likely operates through a distinct mode of action compared to 5-FU. Therefore, the use of compounds such as **Ru-5-FUA**, which combine different mechanisms of action against cancer cells, is a promising approach to overcoming drug resistance. Additionally, it may contribute to the reduction of the dosage of chemotherapy drugs, potentially diminishing their secondary effects and improving the therapeutic outcomes.

## Experimental Section

### Materials

[Ru_2_Cl_2_(DPhF)_3_], [Ru_2_Cl(DPhF)_3_(O_2_CCH_3_)] (**RuA**), and 5-fluorouracil-1-acetic acid (5-FUAH) were prepared as reported elsewhere.^54−56^ The other reactants and solvents were obtained from commercial sources and were used without further purification.

### Characterization of the Compounds

Elemental analyses were carried out by the Microanalytical Service of the Complutense University of Madrid (UCM). Matrix-assisted laser desorption/ionization (MALDI) mass spectra were collected by the Mass Spectrometry Service of UCM using a MALDI TOF/TOF Bruker Ultraflex spectrometer. ^19^F-NMR spectrum was collected by the Magnetic Resonance Service of UCM employing a Bruker AVIII HD 300 MHz BACS-60 spectrometer. FT-IR spectra were recorded by employing a PerkinElmer Spectrum 100 instrument including a universal ATR accessory. Electronic spectra were collected by using a Cary 5G spectrometer.

#### X-ray Diffraction Data Collection and Structure Refinement of **Ru-5-FUA**

A suitable single crystal of **Ru-5-FUA**·0.5THF was measured at 250 K, using a Bruker Kappa Apex II diffractometer with graphite monochromated Mo Kα radiation (λ = 0.71073 Å) from a conventional sealed tube by the Single Crystal X-ray Diffraction Laboratory of Autonomous University of Madrid. The structure was solved using Olex2^57^ with SHELXT using intrinsic phasing^58^ and refined with the SHELXL^59^ using least-squares minimization. Non-hydrogen atoms were refined anisotropically. Hydrogen atoms were included at their calculated positions determined by molecular geometry with fixed isotropic contributions. CCDC 2331581 contains the crystallographic data. More detailed information can be found in the Supporting Information (Tables S1 and S2).

### Characterization of the Nanoparticles

The hydrodynamic size of the polymer particles in water was measured by using a Malvern Zetasizer Nano-ZS instrument (He–Ne laser: 633 nm, scattering angle: 173°, temperature: 25 °C), from the Spectroscopic and Correlation Unit (CAI Chemical Technologies) at UCM. Zeta potential values were measured in water with a dip cell at 25 °C. Transmission electron microscopy (TEM) images were obtained with a JEM 1400 K PLUS instrument operating at 100 kV for nonstained samples, from the National Center for Electron Microscopy at UCM. Samples were prepared by dropping 5 μL of the colloidal suspension on Formvar/carbon-supported copper grids, and the solvent was allowed to evaporate for 24 h. The histogram was calculated from TEM images using the ImageJ software.^60^ Freeze-drying of particles was carried out by using a Telstar Lyoquest lyophilizer. An Elmasonic P 300 H ultrasonic unit (Elma-Hans Schmidbauer GmbH & Co, max. 1580 W, and 80 kHz) was used for redispersion of the particles after lyophilization.

### Synthesis

#### Synthesis of [Ru_2_Cl(DPhF)_3_(5-FUA)] (**Ru-5-FUA**)

A suspension of 200 mg of [Ru_2_Cl_2_(DPhF)_3_] (0.23 mmol) in 32 mL of acetone was added to a solution of 45 mg of 5-fluorouracil-1-acetic acid (0.24 mmol) in 16 mL of water. The purple suspension obtained was stirred for 3 days at room temperature. The mixture was filtered, and part of the solvent was evaporated. Later, dichloromethane/water extraction was carried out. The organic phase was dried over magnesium sulfate and filtered. The solvent was evaporated, and the solid obtained was washed with cold toluene (3 × 5 mL) and dried. Yield: 135 mg (58%). Anal. calcd for Ru_2_ClC_45_H_37_N_8_O_4_F·0.5H_2_O (1019.446 g·mol^–1^): C, 53.02%; H, 3.76%; N, 10.99%. Found: C, 53.27%; H, 3.92%; N, 10.64%. IR: ν̃ (cm^–1^) 3174w, 3059w, 3030w, 2956w, 2839w, 1700m, 1661m, 1592m, 1525vs, 1486vs, 1451m, 1433m, 1404m,1375m, 1315s, 1215vs, 1146m, 1076m, 1026m, 1003m, 976m, 938m, 881m, 800m,776m, 756vs, 693vs, 657m, 601m, 571m. UV/vis/NIR (CH_2_Cl_2_): λ_max_/nm (ε/M^–1^ cm^–1^) 523 (5200), ∼ 570 sh (4800), ∼640 sh (3500). MS (MALDI): *m*/*z* 976.179 (calcd 976; error = 183.40 ppm) [M-Cl]^+^. ^19^F-NMR (300 MHz, DMSO): δ/ppm −168.75 (s, 1F). Single crystals of **Ru-5-FUA**·0.5THF were obtained by slow diffusion of hexane in a THF solution of the complex.

#### Synthesis of Polymer Nanoparticles Loaded with **Ru-5-FUA**

Method 1: A solution of **Ru-5-FUA** (0.5 mg, 4.64 × 10^–4^ mmol) and PMMA (0.5 mg) in 1 mL of THF was slowly added over 10 mL of distilled water under magnetic stirring at 600 rpm, which was maintained stirring for additional 5 min. The oil-in-water droplets were stabilized without stirring for 24 h at 20 °C (or 4 °C). Finally, THF was removed by slow evaporation for 24 h at room temperature (or fast evaporation using a rotary evaporator) to yield the aqueous dispersions of polymer particles containing **Ru-5-FUA**. Cotton filters were used to remove any residual polymer fiber before particle characterization.

Method 2: Polymer particles were obtained as in method 1, but by adding water (10 mL) over the THF solution (1 mL) that contained PMMA (0.5 mg) and **Ru-5-FUA** (0.5 mg, 4.64 × 10^–4^ mmol). The droplet stabilization was carried out for 24 h at 20 °C, and THF was slowly removed during 24 h at room temperature.

#### Synthesis of the Polymer Nanoparticles **PMMA@Ru-5-FUA** Used for Cell Viability Assays

These polymer nanoparticles were prepared via method 1 using ultrapure Milli-Q water, stabilizing the O/W droplets at 20 °C and allowing THF evaporation at room temperature. The obtained polymer particles were freeze-dried for preservation. This synthetic procedure was carried out in triplicate.

Before use, the lyophilized particles from three preparations as described above, which contained 1.5 mg (1.39 × 10^–3^ mmol) of **Ru-5-FUA**, were successfully redispersed in 1 mL of Milli-Q water under ultrasound treatment for 120 min at room temperature ([**Ru-5-FUA**] = 1393.17 μM) and filtered through a membrane with a 0.22 μm pore size. The final concentration of **Ru-5-FUA** was determined by UV–vis spectroscopy using the following equation: *A* (523 nm) = 5131.79696·[**Ru-5-FUA**]. The calibration curve was measured in dichloromethane in the 3 × 10^–6^–4 × 10^–4^ M range.

#### Synthesis of the Polymer Nanoparticles PMMA, **PMMA@C6**, and **PMMA@RuA**

The particles were prepared as the **PMMA@Ru-5-FUA** ones used for cell viability assays but employing only PMMA, PMMA, and coumarin 6 (C6) instead of **Ru-5-FUA**, or PMMA and [Ru_2_Cl(DPhF)_3_(O_2_CCH_3_)] (**RuA**) instead of **Ru-5-FUA**. Particles were used as synthesized in the case of PMMA and **PMMA@C6.** The concentration of C6 in **PMMA@C6** was determined from UV–vis spectroscopy.^61^

**PMMA@RuA** particles of three replicates (containing 1.5 mg of RuA, 1.70 × 10^–3^ mmol) were lyophilized and redispersed in 1 mL of Milli-Q water under ultrasound treatment for 120 min at room temperature. The final concentration of RuA in **PMMA@RuA** was calculated using the following equation: *A* (517 nm) = 3213.65786·[**RuA**]. The calibration curve was measured in dichloromethane solutions in the 4 × 10^–6^–3 × 10^–4^ M range.

### Culture and Cell Viability Assays

Human colon adenocarcinoma Caco-2 cells (ATCC HTB-37) were cultured in DMEM (Dulbecco’s modified Eagle’s medium) containing 4.5 g/L glucose and supplemented with 10% heat-inactivated fetal calf serum, penicillin (50 IU/mL), streptomycin (50 μg/mL) and 2 mM glutamine. Cells were routinely subcultured by trypsinization (0.05% trypsin, 0.02% EDTA) before reaching confluence to avoid self-differentiation.

Concentrated stock solutions of **PMMA@Ru-5-FUA**, **PMMA@RuA**, and PMMA nanoparticles were prepared in Milli-Q water as described above. A concentrated stock solution of 5-FU in DMSO was also prepared. Cell viability assays were carried out in 24-well culture plates (Costar); cells were seeded at 10^4^ cells/cm^2^ and were cultured for 2 days under standard conditions until they reached 40–50% confluence. Then, the medium was replaced by a new one containing increasing concentrations of **PMMA@Ru-5-FUA**, **PMMA@RuA**, PMMA nanoparticles, or 5-FU in a final volume of 500 μL. Controls contained the same amount of Milli-Q water (always less than 20%) or 0.5% DMSO than wells containing the cytotoxic components. After 72 h incubation in the presence of the different agents, cell viability was measured by evaluation of the reduction of 3-[4,5-dimethylthiazol-2-yl]-2,5-diphenyltetrazolium bromide (MTT, Sigma) as described elsewhere.^62^ Briefly after each treatment, cells were washed with PBS to remove ruthenium complexes from the culture media and incubated at 37 °C for 4 h in the dark in the presence of 125 μL of 0.5 mg/mL MTT in PBS. Afterward, MTT was removed, and cell monolayers were carefully washed with PBS and allowed to dry. Then, the dye was extracted in 0.04 M HCl in isopropyl alcohol, and absorbance was measured at 570 nm.

The concentration that inhibits the viability of cells by 50% (IC_50_) compared to untreated control cells was determined from the cell viability curves adjusted by nonlinear regression to a three-parameter exponential decay.

#### Confocal Laser Scanning Microscopy

Caco-2 cells were seeded at 2 × 10^4^ cells/cm^2^ in 24-well plates containing sterile round glass coverslips. After 2 days of incubation under standard conditions to allow cell attachment to the glass plates, cells were exposed for 1, 4, and 24 h to **PMMA@C6** nanoparticles at a final concentration of 7 ng/mL. After exposure, cells were washed using PBS-Tween 20 (0.05%, v/v). Cell-containing coverslips were then mounted on slides using anti fading fluorescent mounting medium (Dako). Samples were analyzed using an Olympus FluoView 1200 microscope from the Fluorescence Microscopy Unit of UCM and processed using ImageJ software.^60^

## Supporting Information Available

Mass spectrum (MALDI) data for **Ru-5-FUA** (Figures S1 and S2); infrared spectrum of **Ru-5-FUA** (Figure S3); electronic spectrum of **Ru-5-FUA** in solution (Figure S4); crystal and structure refinement data for **Ru-5-FUA·0.5THF** (Table S1); selected bond distances (Å) and angles (°) for **Ru-5-FUA·0.5THF** (Table S2); synthetic pathways for the entrapment of **Ru-5-FUA** into PMMA particles (Figure S5); and Z-average and PDI values recorded by DLS for **PMMA@Ru-5-FUA** nanoparticles for three months (Figure S6) (PDF)
