# Supplementary material for: Overcoming Resistance of Caco-2 Cells to 5-Fluorouracil through Diruthenium Complex Encapsulation in PMMA Nanoparticles
Source: Inorg Chem. 2024 Jun 4;63(28):12870–9. doi: 10.1021/acs.inorgchem.4c01323 (PMC11256753; doi:10.1021/acs.inorgchem.4c01323)
Supplement: Supplementary file 1 — ic4c01323_si_001.pdf [file ic4c01323_si_001.pdf]

Overcoming Resistance of Caco-2 Cells to 5-Fluorouracil Through Diruthenium Complex Encapsulation in PMMA Nanoparticles

Isabel Coloma,[1] Jorge Parrón-Ballesteros,[2] Miguel Cortijo,[1] Cristián Cuerva,[1] Javier Turnay,[2] Santiago Herrero[1*]*

[1]MatMoPol Research Group, Inorganic Chemistry Department, Faculty of Chemical Sciences, Complutense University of Madrid, E-28040 Madrid, Spain.

[2]Department of Biochemistry and Molecular Biology, Faculty of Chemical Sciences, Complutense University of Madrid, E-28040 Madrid, Spain.

INDEX

- Figure S1. Mass spectrum (MALDI) of **Ru-5-FUA**.
- Figure S2. Enlargement of the peak corresponding to the base peak of the mass spectrum of **Ru-5-FUA**.
- Figure S3. Infrared spectrum of **Ru-5-FUA**.
- Figure S4. Electronic spectrum of **Ru-5-FUA** in dichloromethane solution (~10^{-4} M).
- Table S1. Crystal and structure refinement data for **Ru-5-FUA**·0.5THF.
- Table S2. Selected bond distances (Å) and angles (deg) for **Ru-5-FUA**·0.5THF.
- Figure S5. Synthetic pathways for the entrapment of **Ru-5-FUA** into PMMA particles.
- Figure S6. Z-average and PDI values recorded by DLS for **PMMA@Ru-5-FUA** nanoparticles for three months.

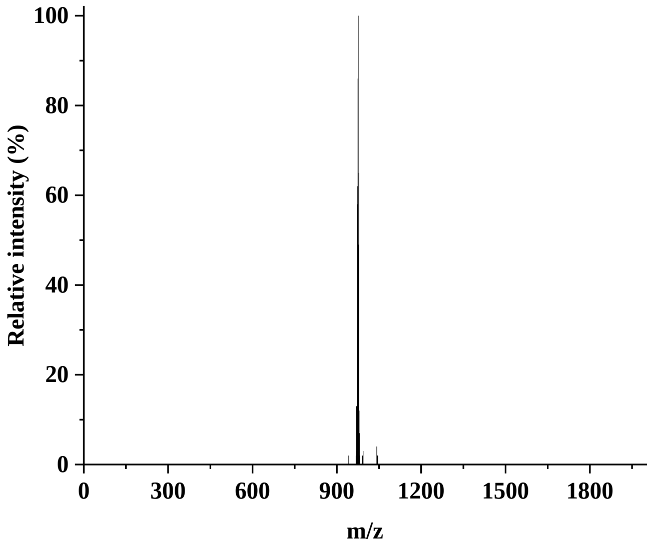

Figure S1. Mass spectrum (MALDI) of **Ru-5-FUA**.

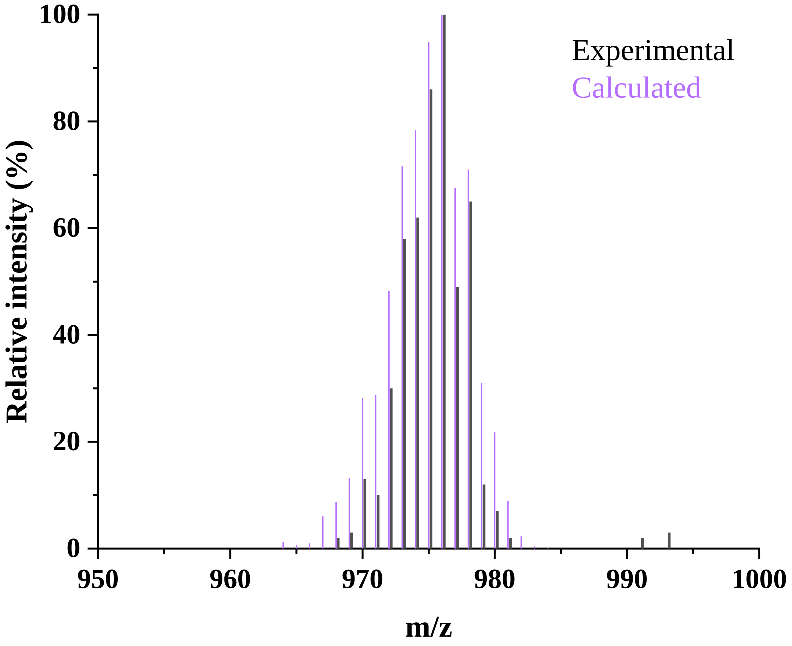

Figure S2. Experimental (black) and calculated (purple) isotopic distribution of $[M\text{-}Cl]^+$ peak.

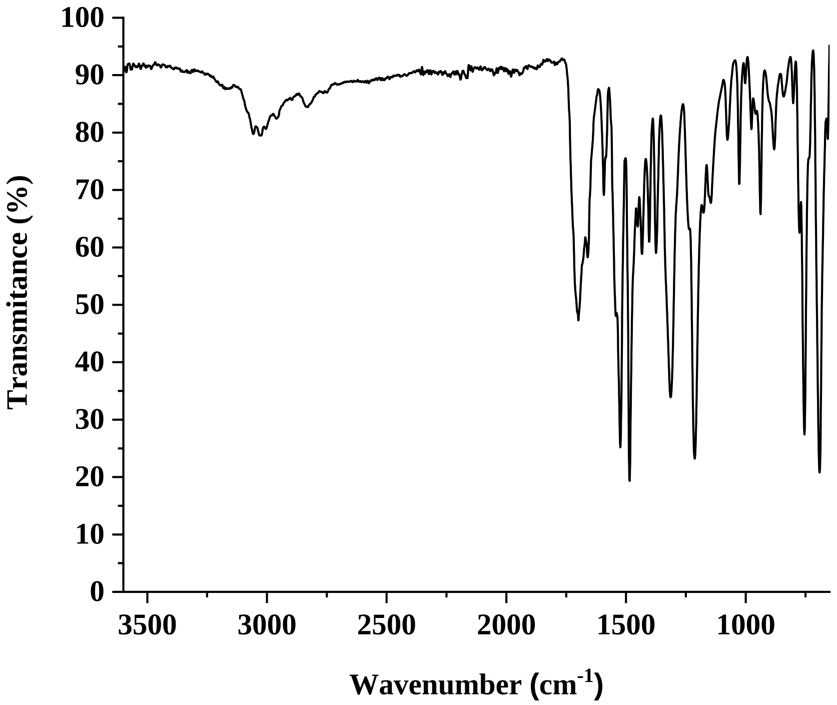

Figure S3. Infrared spectrum of **Ru-5-FUA**.

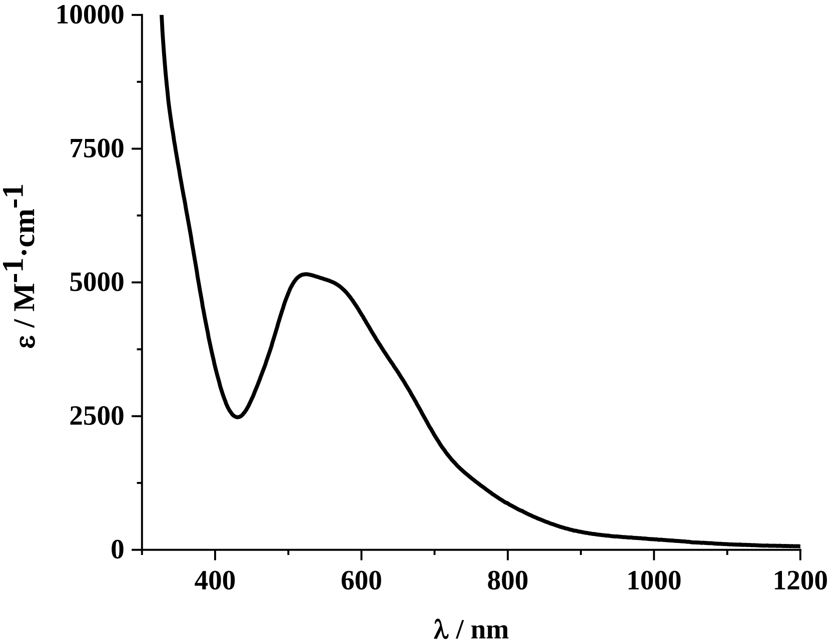

Figure S4. Electronic spectrum of **Ru-5-FUA** in dichloromethane solution (~10^{-4} M).

Table S1. Crystal and structure refinement data for **Ru-5-FUA**·0.5THF.

**Empirical formula**	$C_{47}H_{41}ClFN_8O_{4.5}Ru_2$
**Formula weight**	1046.47
**Temperature/K**	250
**Crystal system**	Monoclinic
**Space group**	C2/c
**a/Å**	19.3683(6)
**b/Å**	16.6533(5)
**c/Å**	27.9732(11)
**α/°**	90
**β/°**	91.6070(10)
**γ/°**	90
**Volume/Å^3**	9019.1 (5)
**Z**	8
**ρ_{calc}g/cm^3**	1.541
**μ/mm^{-1}**	0.788
**F(000)**	4232.0
**Crystal size/mm^3**	0.198 x 0.197 x 0.027
**Radiation**	MoKα (λ = 0.71073)
**2Θ range for data collection/°**	4.892 to 50.7
**Index ranges**	-23 ≤ h ≤ 22, -20 ≤ k ≤ 20, -33 ≤ l ≤ 33
**Reflections collected**	51010
**Independent reflections**	8263 [R_{int} = 0.0610, R_{sigma} = 0.0485]
**Data/restraints/parameters**	8263/ 0 / 573
**Goodness-of-fit on F^2**	0.947
**Final R indexes [I>=2σ (I)]**	R_1 = 0.0335, wR_2 = 0.0672
**Final R indexes [all data]**	R_1 = 0.0562, wR_2 = 0.0754
**Largest diff. peak/hole / e Å^{-3}**	0.35/-0.28

Table S2. Selected bond distances (Å) and angles (deg) for **Ru-5-FUA**·0.5THF.

**Ru1**	**Ru2**	2.3124(3)	**Cl1**	**Ru1**	**Ru2**	177.66(2)
**Ru1**	**Cl1**	2.3953(8)	**O1**	**Ru1**	**N5**	176.75(9)
**Ru1**	**O1**	2.088(2)	**O2**	**Ru2**	**N6**	178.4(1)
**Ru2**	**O2**	2.062(2)	**N3**	**Ru1**	**N7**	173.1(1)
**Ru1**	**N3**	2.092(3)	**N4**	**Ru2**	**N8**	175.2(1)
**Ru2**	**N4**	2.022(3)				
**Ru1**	**N5**	2.041(3)				
**Ru2**	**N6**	2.009(3)				
**Ru1**	**N7**	2.087(2)				
**Ru2**	**N8**	2.041(3)				

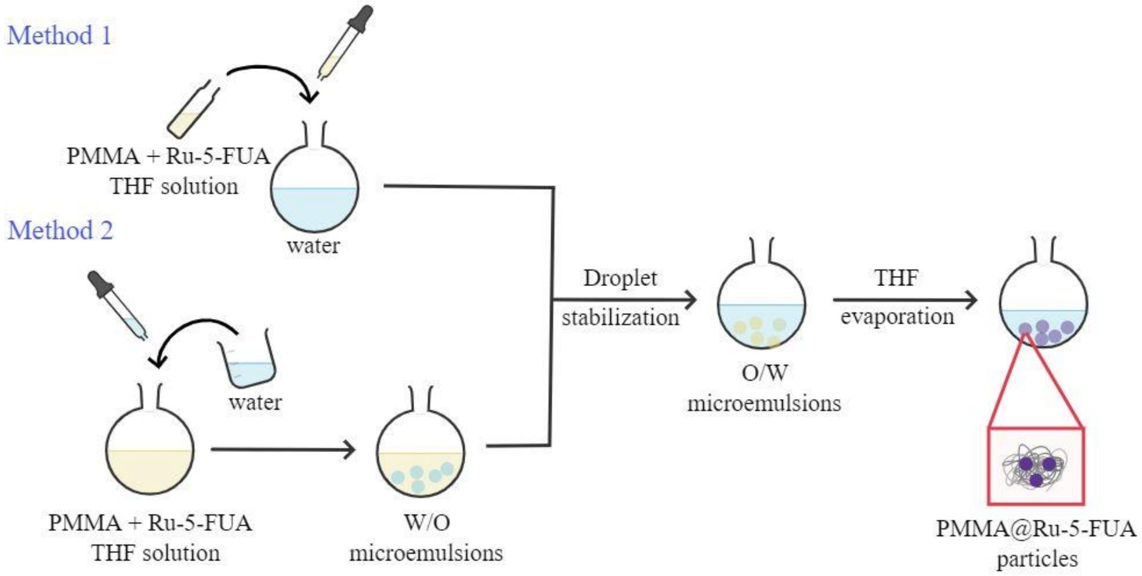

Figure S5. Synthetic pathways for the entrapment of **Ru-5-FUA** into PMMA particles.

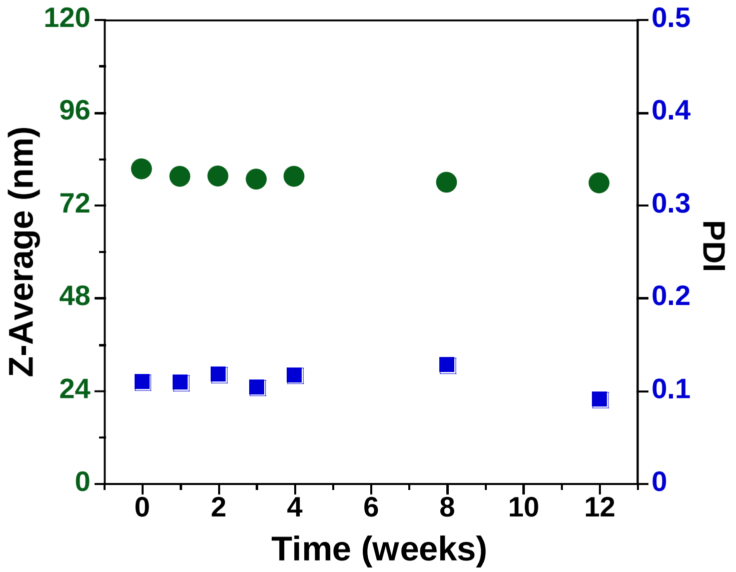

Figure S6. Z-average and PDI values recorded by DLS for **PMMA@Ru-5-FUA** nanoparticles for three months.
